# Exploration of risk factors for hemoglobinuria and acute kidney injury following iliofemoral venous mechanical thrombectomy

**DOI:** 10.1016/j.jimed.2022.10.005

**Published:** 2022-11-09

**Authors:** Xinqiang Han, Qingqing Zhang, Fengfei Xia, Yongzhen Zhang, Wenming Wang

**Affiliations:** a Department of Interventional Medicine and Vascular, Binzhou Medical University Hospital, Binzhou, 256603, Shandong, China; b Department of Cerebrovascular, Binzhou Medical University Hospital, Binzhou, 256603, Shandong, China; c Department of Interventional Medicine and Vascular, Binzhou Peoples Hospital, Binzhou, 256600, Shandong, China

**Keywords:** Iliofemoral deep venous thrombosis, Acute kidney injury, Hemoglobinuria, Thrombectomy, Thrombolysis

## Abstract

**Objective:**

To evaluate the risk factors for hemoglobinuria and acute kidney injury (AKI) after percutaneous mechanical thrombectomy (MT) with or without catheter-directed thrombolysis (CDT) for iliofemoral deep vein thrombosis (IFDVT).

**Methods:**

Patients with IFDVT who had MT with the AngioJet catheter (group A), MT plus CDT (group B), or CDT alone (group C) from January 2016 to March 2020 were retrospectively evaluated. Hemoglobinuria was monitored throughout the treatment course, and postoperative AKI was assessed by comparing the preoperative (baseline) and postoperative serum creatinine (sCr) levels from the electronic medical records of all patients. AKI was defined as an elevation in the sCr level exceeding 26.5 ​μmol/L within 72 ​h after the operation according to the Kidney Disease Improving Global Outcomes criteria.

**Results:**

A total of 493 consecutive patients with IFDVT were reviewed, of which 382 (mean age, 56 ​± ​11 years; 41% of them were females; 97 in group A, 128 in group B, and 157 in group C) were finally analyzed. Macroscopic hemoglobinuria was evident in 44.89% of the patients of the MT groups (101/225, 39 in group A, and 62 in group B), with no significant difference between the groups (P ​= ​0.219), but not in the patients in group C. None of the patients developed AKI (mean sCr difference −2.76 ​± ​13.80 ​μmol/L, range ​= ​−80.20 to 20.60 ​μmol/L) within 72 ​h after surgery.

**Conclusions:**

Rheolytic MT is an independent risk factor for hemoglobinuria. A proper aspiration strategy, hydration, and alkalization following thrombectomy are particularly favorable for preventing AKI.

## 1 Introduction

Deep vein thrombosis (DVT) is a common clinical disease estimated to affect 0.1% of the population worldwide. It can result in serious complications without prompt and adequate diagnosis and treatment.^1^ Early endovascular treatment for clot clearance, especially for iliofemoral deep vein thrombosis (IFDVT), has been shown to significantly reduce or eliminate the incidence of post-thrombotic syndrome (PTS) and pulmonary embolism (PE).^2^ Previous guidelines have recommended endovascular treatment, including pharmacomechanical thrombectomy (PMT), mechanical thrombectomy (MT), or catheter-directed thrombolysis (CDT), as the first-line strategy for acute IFDVT.^3^, ^4^, ^5^ Rheolytic MT using the AngioJet device is the better choice for early thrombus removal and combines the advantages of pharmacological thrombolysis and MT to rapidly reduce thrombus burden and restore venous patency. This has additional advantages over CDT, such as shorter inpatient treatment time and fewer bleeding complications.^6^, ^7^, ^8^

Hemoglobinuria and intravascular hemolysis during the MT procedure have garnered increasing attention from clinicians in recent years.^9^, ^10^, ^11^, ^12^, ^13^ High-pressure forcible sprays cause shear stress at the working site during the process of MT, which has the potential to cause mechanical damage to circulating red blood cells (RBCs), resulting in intravascular hemolysis and gross hemoglobinuria. Additionally, there is potential impairment of renal function, which may secondarily develop into acute kidney injury (AKI).^11^^,^^14^

However, although hemolysis induced by MT has already been well described in the literature,^9^, ^10^, ^11^, ^12^^,^^14^^,^^15^ the incidence of AKI following MT varies widely due to the different criteria used to define it in the literature.^9^, ^10^, ^11^, ^12^ To our knowledge, since 2004, there have been at least three reported proposals to diagnose AKI depending on changes in serum creatinine (sCr) levels or urine output: the Risk, Injury, Failure, Loss, and End-Stage Renal Disease (RIFLE) criteria; the AKI Network (AKIN) criteria; and the Kidney Disease Improving Global Outcomes (KDIGO) criteria.^16^, ^17^, ^18^ Of these three, the KDIGO criteria are the most commonly used diagnostic standards in routine clinical practice due to their feasibility and easy application.^19^, ^20^, ^21^ In addition, perioperative management strategies might also have an impact on the occurrence of AKI.

So, we describe our novel perioperative management strategies and retrospectively review our experience in patients with IFDVT who underwent MT to determine the risk factors for hemoglobinuria and renal complications.

## 2 Materials and methods

### 2.1 Patients and ethics statement

This retrospective cohort study included 493 consecutive patients with acute DVT who underwent interventional procedures between January 2016 and March 2020 and met the inclusion criteria. Among these, 382 patients were selected for the final study based on the inclusion and exclusion criteria. The included patients were divided into three groups: group A (MT), group B (MT plus CDT), and group C (CDT-only). This study was conducted in accordance with the Declaration of Helsinki for human research (as revised in 2013) and was approved by the ethics committee of our hospital (2020 Ethics Approval No. LW-026). The requirement for individual consent for this retrospective study was waived.

#### 2.1.1 Inclusion criteria

The inclusion criteria were as follows: (i) acute unilateral DVT involving the iliofemoral vein segment or vena cava segment; (ii) one or more baseline sCr results within a 24-h window prior to surgery; and (iii) one or more postoperative sCr results obtained 72 ​h after interventional procedures.

#### 2.1.2 Exclusion criteria

Patients were excluded from this study if they met at least one of the following exclusion criteria: (i) bilateral DVT or no involvement of the iliofemoral segment; (ii) preoperative AKI or history of end-stage kidney disease or chronic kidney disease (CKD) at hospital admission; (iii) history of nephrectomy; (iv) lack of pre or postoperative sCr results or incomplete clinical data; (v) pregnancy; (vi) age younger than 14 years or older than 80 years; and (vii) contraindications to anticoagulation and thrombolysis.

### 2.2 Study design

Clinical data related to the treatment process, including preoperative (baseline) and postoperative laboratory values such as levels of sCr, hemoglobin (Hb), hematocrit (HCT), and treatment modality, were obtained from electronic medical records. Additionally, complete postoperative surveillance data such as urine output and color per patient were included. The baseline value was the result of the most recent preoperative examination that was done less than 24 ​h before the procedure.

Currently, there are no consensus criteria for the diagnosis of hemolysis-related AKI. We used a more common and feasible clinical definition for consistency with current clinical guidelines (KDIGO 2012).^22^ The sCr level at less than 72 ​h after the operation was selected for additional analysis and to determine the AKI incidence. AKI was defined as an increase in the sCr level of more than 26.5 ​μmol/L at 72 ​h postoperatively. The worst postoperative laboratory values (e.g., the highest value of sCr) within this period were selected for subsequent analysis.^10^ The changes in sCr, potassium, Hb, and HCT levels in each patient were calculated by comparing the corresponding baseline values with the worst laboratory values during the 72-h window of observation. All sCr assays were standardized using isotope dilution mass spectrometry.

### 2.3 Endovascular therapeutic process

Endovascular interventions were mainly determined at the discretion of the interventional radiologist's skill rather than by the protocol. On safety grounds, a retrievable inferior vena cava (IVC) filter (Aegisy, Lifetech Scientific, SZ, China) was implanted through a contralateral femoral venous approach to prevent fatal PE according to our institutional guidelines and national guidelines.^23^ Percutaneous transluminal angioplasty (PTA) and stent placement were performed when iliocaval outflow compression or stenosis was greater than 50% after the PMT or CDT procedure. Regarding the anticoagulant regimen after discharge, rivaroxaban 20 ​mg/day was administered for at least three to six months and much longer in some cases.

#### 2.3.1 MT groups

For the femoral vein, popliteal vein, or anterior tibial vein approach, a 0.035-inch guidewire (Terumo, Tokyo, Japan) was introduced in a 6 Fr AngioJet thrombus aspiration catheter (Boston Scientific, Marlborough, MA, USA) through the thrombosed vein segment. An AngioJet catheter was used to perform MT (working in a thrombus aspiration model). This procedure was repeated until the thrombus was aspirated to the greatest extent possible, with a maximum time of 480 ​s. At the end of the MT procedure, angiography was used to measure the thrombus clearance rates (grade III: 95% thrombus removal; grade II: 50–94% thrombus removal; and grade I: 50% thrombus removal). CDT was performed if the angiographically visible residual thrombus was >50% after PMT. All of the patients underwent urine alkalization, hydration and hepatorenal function and routine blood tests were monitored postoperatively every 24 ​h for at least three days.

#### 2.3.2 CDT group

For the anterior tibial vein, popliteal vein, or femoral vein approach, a thrombolytic eluting catheter with multiple side holes and different lengths (UniFuse catheter; AngioDynamics, Queensbury, NY, USA) was placed into the thrombosed venous segment. The urokinase (50 ​mL of normal saline ​+ ​250,000 units per 8–12 ​h) was injected through the catheter *via* a micropump at a total dose of 1,000,000–3,500,000 units, with variations from one individual to another. Unfractionated heparin was administered at 300–500 IU per hour *via* a thrombolytic catheter or access sheath. Every four to 6 ​h, the fibrinogen levels and activated partial thromboplastin time (APTT) were used to evaluate the blood coagulation. The APTT was adjusted to prolongation by 1.2–1.5-fold, and the urokinase dose was halved when the fibrinogen level dropped below 1.5 ​g/L. Routine blood examinations were performed daily. Unfractionated heparin was discontinued when the platelet count declined to less than 50% of the baseline value. At the same time, heparin-induced thrombocytopenia was considered and further confirmed. A venogram was repeated every 48–72 ​h to assess the effect of thrombolysis and adjust the thrombolytic catheter position. Thrombolysis was stopped if any of the following conditions occur (i) fibrinogen level less than 1.0 ​g/L; (ii) major hemorrhagic complications or catheter-related infections; (iii) complete lysis of the thrombus; and (iv) no change in the extent of the thrombus on two successive venography assessments.^24^

#### 2.3.3 Perioperative management

All the patients underwent urethral catheterization 1 ​h before the interventional procedure. The nephroprotective measures were as follows: normal saline was administered intravenously to each patient at 1.5 ​mL/kg/h from two to 6 ​h before the endovascular treatment to 24 ​h after the procedure.^10^ Subsequently, 5% sodium bicarbonate at 2.5 ​mmol/kg was transfused half an hour before MT to alkalize urine, and the input was determined by total primary urine output and urine color.

The maximum dose of suction fluid used in the MT process on every patient was 500 ​mL. No other nephrotoxic drugs were used during hospitalization, except for the contrast agent (the dosage was not recorded in the electronic medical record). There were no differences in the approach to nephroprotective interventions among the groups.

Hematuria was determined by visual inspection and urine occult blood test results. Changes in urine output and color were also recorded. All patients who underwent MT were divided into two subgroups based on the presence or absence of gross hematuria postoperatively. Routine blood, hepatorenal function, and urine tests were conducted every 24 ​h postoperatively, and any changes in these parameters were identified.

### 2.4 Statistical analysis

Statistical analysis was performed using IBM SPSS Statistics for Windows, version 23 (IBM Corp., Armonk, N.Y., USA). Continuous variables are presented as the mean ​± ​standard deviation. The Kruskal–Wallis test, one-way analysis, Mann–Whitney *U* test, paired *t*-test, or unpaired *t*-test were used to compare continuous data, and the χ^2^ test was used to compare categorical data. The statistical significance was set at P ​< ​0.05.

## 3 Results

### 3.1 Patient baseline characteristics

A total of 493 consecutive patients diagnosed with DVT were enrolled in the study. Thirty-seven patients with incomplete clinical data, 59 without IFDVT, and 15 with bilateral DVT were excluded from the study. The remaining 382 individuals with confirmed IFDVT were included in the final analysis. Based on the treatment procedure, 97, 128, and 157 of these patients were assigned to groups A, B, and C, respectively.

The baseline demographics and clinical characteristics are presented in Table 1. There were no significant differences in the baseline data among the three groups (Table 2). The average baseline sCr values were similar among the three groups (P ​= ​0.092). Only seven patients in group B (5%) and one patient in group C (0.6%) had a baseline sCr level >132 ​μmol/L (1.5 ​mg/dL) before the procedures.

**Table 1:** Baseline patients characteristics (n ​= ​382).

Characteristics	Value
Age (years), mean ​± ​SD	56 ​± ​11
Sex	
Female, n (%)	158(41%)
Male, n (%)	224(59%)
Duration of symptoms (days), mean ​± ​SD	4.9 ​± ​3.1
1～7, n (%)	317(83%)
8～14, n (%)	65(17%)
Thrombus location	
Left limb, n (%)	287(75%)
Right limb, n (%)	95(25%)

**Table 2:** Comparison of baseline characteristics between groups.

Characteristics	PMT Group	PMT ​+ ​CDT Group	CDT Group	P value	χ^2^ value
	(n ​= ​97)	(n ​= ​128)	(n ​= ​157)		
Age(years), mean ​± ​SD	56.9 ​± ​13.3	57.0 ​± ​11.1	54.8 ​± ​10.2	0.162	3.636
**Gender**					
Female, n (%)	39 (40%)	58 (45%)	61 (39%)	0.526	1.284
**Comorbidities**					
Hypertension, n (%)	53 (55%)	79 (62%)	76 (48%)	0.080	5.039
Diabetes, n (%)	59 (61%)	82 (64%)	82 (52%)	0.112	4.383
Baseline sCr value	67.5 ​± ​21.2	71.7 ​± ​32.0	74.3 ​± ​27.1	0.092	4.782
(μmol/L), mean ​± ​SD					
Baseline HCT value	44.2 ​± ​5.3	43.6 ​± ​5.5	44.4 ​± ​4.0	0.673	0.791
(%), mean ​± ​SD					
**Thrombus extent**				0.110	4.417
Iliac-proximal femoral vein, n (%)	51(53%)	85(66%)	94(60%)		
Iliofemoral-popliteal vein, n (%)	46(47%)	43(34%)	63(40%)		

### 3.2 Hemoglobinuria and PMT using AngioJet

The MT group exhibited more macroscopic hemoglobinuria (44.89%, 39 patients in group A and 62 in group B) regardless of whether MT was combined with adjuvant CDT (χ^2^ ​= ​1.511, P ​= ​0.219), whereas the CDT-only group did not.

The differences between the subgroups that did and did not exhibit hemoglobinuria after the MT procedure were compared. The findings are summarized as follows: 1) The occurrence of hemoglobinuria was determined by the AngioJet catheter's exposure to blood flow or by the timing of recanalization of the occluded common iliac vein. 2) Regarding the duration of the MT procedure, there was no significant difference in the duration of thrombus aspiration between the two groups. 3) Regarding the extent of the thrombus, the iliac-proximal femoral and distal femoral-popliteal veins (including trifurcation) were considered independent anatomic sites. There was no significant difference in the thrombus load between the two groups (Table 3).

**Table 3:** Comparison of characteristics between hemoglobinuria group and no hemoglobinuria group.

Characteristics	Hemoglobinuria group	No hemoglobinuria group	P value
	(n ​= ​101)	(n ​= ​124)	
**Exposure ​to ​blood ​flow early**			<0.001
Yes, n (%)	69(68%)	15(12%)	
No, n (%)	32(32%)	109(88%)	
**Duration of PMT** (s), mean ​± ​SD	368 ​± ​102	386 ​± ​81	0.511
**Extent of thrombosis**			0.574
Iliac-proximal femoral vein, n (%)	42(42%)	47(38%)	
Iliofemoral-popliteal vein, n (%)	59(58%)	77(62%)	
**CDT combined**			0.219
Yes, n (%)	62(61%)	66(53%)	
No, n (%)	39(39%)	58(47%)	

### 3.3 Clinical outcomes

A total of 225 patients underwent MT (97 in group A and 128 in group B), and the success rate was 100%. Thrombus clearance rates were grade III in 91 patients, grade II in 117 patients, and grade I in 17 patients. Approximately 4% (15/382) of the IVC filters were not retrieved, and around 2% (7/382) of the patients were diagnosed with symptomatic PE. The duration of thrombus aspiration ranged from 119 to 480 ​s (mean, 378 ​± ​92 ​s).

Hemolysis was diagnosed based on the occurrence of hemoglobinuria and decreased HCT levels. The changes in the levels of Hb and HCT between the preoperative baseline and the minimum values within 72 ​h postoperatively in each group were significantly different (P ​< ​0.05). The difference between postoperative and preoperative Hb was −31 to 8 ​g/L (mean, −4.4 ​± ​5.0), and no blood transfusion was needed (the transfusion threshold was set to Hb ​< ​7 ​g/L) (Table 4).

**Table 4:** Comparison of creatine, RBC, hematocrit and potassium levels pre- and post-procedure.

Characteristics	PMT Group	PMT ​+ ​CDT Group	CDT Group	P value
	mean ​± ​SD, (n ​= ​97)	mean ​± ​SD, (n ​= ​128)	mean ​± ​SD, (n ​= ​157)	
**Creatinine** (μmol/L)				
pre OP^†^	67.5 ​± ​21.2	71.7 ​± ​32.0	74.3 ​± ​27.1	
post OP^†^	65.4 ​± ​21.0	62.9 ​± ​28.1	76.0 ​± ​25.8	
**Hemoglobin** (g/L)				
pre OP	132.4 ​± ​16.1	132.2 ​± ​17.0	131.3 ​± ​11.5	
post OP	127.9 ​± ​15.9	125.8 ​± ​17.1	128.5 ​± ​11.2	
*difference*^a^	−4.5 ​± ​5.0	−6.2 ​± ​5.9	−2.9 ​± ​3.1	<0.001
**Hematocrit** (%)				
pre OP	44.2 ​± ​5.3	43.6 ​± ​5.5	44.4 ​± ​4.0	
post OP	43.0 ​± ​5.5	42.2 ​± ​5.7	43.7 ​± ​4.0	
*difference*	−1.2 ​± ​2.3	−1.5 ​± ​2.3	−0.7 ​± ​1.1	0.001
**Potassium** (mmol/L)				
pre OP	4.27 ​± ​0.58	4.21 ​± ​0.60	4.32 ​± ​0.61	
post OP	3.95 ​± ​0.62	3.92 ​± ​0.54	4.16 ​± ​0.50	

^†^ Pre OP ​= ​preoperative.

^†^ post OP ​= ​postoperative.

a *difference* ​= ​post OP values *minus* pre OP values.

The postoperative urine output ranged from 3200 to 8700 ​mL (mean volume, 5870 ​± ​1399 ​mL) within 24 ​h. The urine color returned to normal in all patients within 24 ​h following the procedure. None of the patients developed AKI as defined by the KDIGO criteria (mean sCr difference, −2.76 ​± ​13.80 ​μmol/L; range, −80.20 to 20.60 ​μmol/L). All patients were monitored for 3–10 days after the procedure, and none of them were oliguric.

After hospital discharge, all patients underwent periodic ultrasound evaluations of the lower extremity venous system and renal function. Among the patients, the longest and shortest follow**-**ups were 36 and six months, respectively, and 11 patients were lost to follow-up. No abnormal renal function was observed during the follow-up period.

## 4 Discussion

This retrospective study demonstrated that percutaneous MT with an AngioJet aspiration catheter for IFDVT is not associated with an increased risk of AKI in a cohort of hospitalized patients treated with appropriate nephroprotective measures by skilled physicians. However, it is undeniable that compared with CDT, MT is strongly associated with hemoglobinuria, especially when AngioJet aspiration catheters are exposed to blood flow, which is associated with a potential hazard to kidney function and may lead to the development of AKI. The major conclusions of our current study were distinguished from those of previous studies in terms of the incidence of AKI.^9^, ^10^, ^11^^,^^15^^,^^25^ However, as this was a retrospective non-randomized study, some unexpected biases may have occurred.

From what is known thus far, AKI is a common and complex clinical condition with high morbidity and mortality,^26^ and there is no consensus on a uniformly accepted definition of AKI, leading to large variability in the reported results.^11^^,^^16^^,^^21^^,^^27^

MT and CDT are the most common treatments for acute IFDVT. To our knowledge, MT has a stronger clearance ability for acute IFDVT than CDT. All of these MT devices can cause intravascular hemolysis, which raises the levels of plasma-free Hb and may increase the risk of developing hemoglobinuria. Massive acute hemoglobinuria, such as paroxysmal nocturnal hemoglobinuria (PNH), post-cardiopulmonary bypass, or glucose-6-phosphate dehydrogenase deficiency, is a well-known cause of AKI.^28^, ^29^, ^30^ Additionally, the mechanisms of plasma heme-induced renal toxicity, such as tubular cast formation, heme-induced intrarenal vasoconstriction, and cytotoxicity, have been extensively studied,^31^ and the severity of cell damage tends to correlate positively with the duration of blood exposure.^32^

Several previous studies have reported the incidence of MT procedure-induced hemolysis and hemoglobinuria, which are recognized as contributors to postoperative renal injury.^15^^,^^33^ However, there are few reported cases in the literature in which the CDT procedure appears to be associated with renal dysfunction. Currently, we do not have a good understanding of why the CDT procedure causes renal impairment, which has been reported previously.^10^^,^^33^ We consider that this may be related to worsening baseline renal function, comorbidities, and the dose of contrast agent.

Notably, the incidence of macroscopic hemoglobinuria was very high after the MT procedure, reaching 44.89% (101/225) in the current study. We took various measures for kidney protection, such as avoiding nephrotoxic agents except for iodinated contrast media, urine alkalinization,^34^ optimizations of perioperative fluid management, diuresis, and close monitoring of urine color, urine output, and kidney function. None of the patients developed AKI.

Interestingly, patients with increased blood flow at the site of thrombus aspiration developed hemoglobinuria soon after the MT procedure. Moreover, the urine progressively darkened with a longer operation time. This observation suggests that hemoglobinuria may be related to the blood flow around the aspirated thrombus and the starting position of the AngioJet aspiration catheter, which has been demonstrated in animal models.^13^ Improvements in technique, namely, the popliteal vein or anterior tibial vein approach, were selected to allow the AngioJet catheter to proceed into the thrombus in a retrograde manner and be exposed to blood flow at the final moments of iliocaval venous occlusion to recanalize at the final time point of the MT process. In theory, this approach can greatly reduce the constant exposure of RBCs to rheology-mediated shear stress, consequently preventing the rapid release of lysed RBC products into the systemic circulation within a short time frame,^35^ and may therefore decrease the occurrence of hemoglobinuria.^24^

Thrombus aspiration duration may affect, to some extent, the occurrence of hemoglobinuria. Arslan et al.^35^ showed that prolongation of the MT operation time leads to the occurrence of massive hemolysis and the development of AKI. Margheri et al.^36^ showed that shortening the duration of thrombus aspiration reduces the probability of developing AKI. Nazarian et al.^37^ found that there was an exponential rather than a linear correlation between hemoglobinuria and aspiration time. These results suggest that control of aspiration time is crucial to decreasing hemoglobinuria.

Based on our clinical experience, there is some information for the prevention of hemoglobinuria and AKI as follows: 1) to minimize the blood flow around the thrombus as much as possible, the AngioJet catheter would contact blood flow only at the final time point of the MT procedure; 2) the duration of thrombus aspiration must be controlled, and angiography should be repeated to assess the presence of old thrombi; 3) adequate and timely hydration and urine alkalization should be carried out; and 4) a full preoperative evaluation, close monitoring of postoperative renal function, and earlier symptomatic treatment are required.

## 5 Conclusion

In summary, in addition to efficacy, the risk of potential renal function impairment should be considered when selecting the most appropriate treatment modality for acute IFDVT. Moreover, it is a long-held belief that the extent of hemoglobinuria is an important factor associated with the development of kidney dysfunction. The risk of renal impairment due to MT could be lowered with the appropriate nephroprotective measures, optimization of operational skills, and perioperative management.

## Contributions

Wenming Wang: Conceptualization, Methodology, Writing- Reviewing and Editing. Xinqiang Han and Qingqing Zhang: Data curation, Writing- Original draft preparation and Funding acquisition. Fengfei Xia and Yongzhen Zhang: Project administration; Resources and Formal analysis.

## Declaration of competing interest

The authors declare that we do not have any commercial or associative interest that represents a conflict of interest in connection with the study submitted.
